# Tracheostomy decannulation methods and procedures in adults: a systematic scoping review protocol

**DOI:** 10.1186/s13643-017-0634-0

**Published:** 2017-12-04

**Authors:** John Kutsukutsa, Tivani Phosa Mashamba-Thompson, Yougan Saman

**Affiliations:** 1 Department of Otorhinolaryngology Head & Neck Surgery, Nelson R Mandela School of Medicine, University of KwaZulu-Natal, 5th Floor, Room 550 Main Building, Durban, 4001 South Africa; 2 Discipline of Public Health, University of KwaZulu-Natal, Durban, 4001 South Africa; 3 Department of Otorhinolaryngology Head & Neck Surgery, University of KwaZulu-Natal, Durban, 4001 South Africa

**Keywords:** Tracheostomy decannulation, Weaning, Methods and procedures

## Abstract

**Background:**

The indications for and the number of tracheostomy procedures has increased with advances in critical care. Studies are indicating likely continued increase in number of tracheostomies. Despite the important benefits of a tracheostomy, its presence is associated with adverse health complications and lowered patient quality of life. Hence, it must be decannulated as soon as it is no longer indicated in a safe and effective manner. There is, however, no agreed universal standard of care for tracheostomy decannulation (TD) in adults. The aims of our study are to systematically map the literature on the decannulation process, reveal knowledge gaps and inform further research.

**Methods:**

The search strategy of this systematic scoping review will involve the following electronic databases: PubMed/MEDLINE, Google Scholar, Union Catalogue of Theses and Dissertations (UCTD) via SABINET Online and WorldCat Dissertations and Theses via OCLC. Articles will also be searched through the “Cited by” search as well as citations included in the reference lists of included articles. Studies from the databases will be title screened and duplicates removed followed by a parallel two-independent reviewer screening of abstracts followed by full articles of selected studies both guided by eligibility criteria. We will extract data from the included studies and the emerging themes will be analysed. The relationship of the emerging themes to the research question will be critically examined. The quality of the included studies will be determined by Mixed Method Appraisal Tool (MMAT). We will use NVIVO version 10 to extract the relevant outcomes and thematic analysis of the studies.

**Discussion:**

We anticipate to find studies that highlight evidence and preference as well as acceptability of TD methods and procedures. We hope to expose knowledge gaps and inform future research. Findings will be disseminated electronically, in print and through peer presentation, conferences and congresses.

**Systematic review registration:**

Our systematic review has been registered in PROSPERO: CRD42017072050.

## Background

The indications for tracheostomies have expanded and the rate at which the procedure carried out has also increased with the advancement of critical care [1–4]. It is estimated that up to 10% of intensive care unit (ICU) patients will require a tracheostomy [5]. This is in addition to the tracheostomies done by various surgical specialities outside of critical care. Although timing of tracheostomy in ICU is still debated [6, 7], it is leaning towards early tracheostomy spurred on by some studies showing benefits of early tracheostomy [8]. In spite of this increase, there is no consensus on the standard approach to its reversal (tracheostomy decannulation) thereby relegating decisions to expert opinion and institutional protocols [9–11]. In a recent survey, non ENT health professionals involved in airway care had a low level of self-rated comfort with tracheostomy tube care [12]. All these may imply patients being exposed to non-scientific, risky decannulation practises or finding themselves under the care of non-ENT health care workers who may not feel comfortable decannulating them.

Article 26 of the United Nations Convention on the Rights of Persons with Disabilities obliges member states to ‘take effective and appropriate measures, including through peer support, to enable persons with disabilities to attain and maintain maximum independence, full physical, mental, social and vocational ability, and full inclusion and participation in all aspects of life’ [13]. Regaining verbal communication is one of the benefits of TD which allows attainment of these goals. Decannulation improves patient comfort, perceived physical appearance in addition to improved speech and swallowing [14–17]. It is therefore prudent to have tracheostomy decannulation (TD) as soon as it is no longer indicated in a safe and effective manner.

Tracheostomy decannulation has a risk of failure with fatal consequences if not managed appropriately. Experts in different settings have different opinions and approaches to TD [18]. This raises the question of whether we understand what is available well enough to design and carry out further research that speaks to all. It is our contention that this systematic scoping review will better our understanding of the decannulation process, expose knowledge gaps and stimulate research to fill in the gaps. We therefore aim to explore evidence on methods and procedures for tracheostomy decannulation in adults. Our objectives are the following:

- ➢ To highlight evidence base for the different methods and procedures for TD
- ➢ To determine the preference of method and procedures for TD in adults
- ➢ To determine the acceptability (to patients and health care workers) of method and procedures for TD in adults

Systematically mapping the available evidence for TD methods and procedures will cascade into better outcomes for tracheostomy patients through enhanced understanding and more scientific approaches to TD.

## Methodology

### Systematic scoping review

We will conduct a systematic scoping review of peer-reviewed and grey literature on the methods and procedures of TD in adults. The review will include a quality assessment. This review will be guided by Arksey and O’Malley’s [19] scoping review framework which stipulates the following steps:

1. Identifying the research question
2. Identifying relevant studies
3. Study selection
4. Charting the data
5. Collating, summarising and reporting the results

### Identifying the research question

The research question is, what is the evidence available for the different methods and procedures for TD in adults?

The sub research questions are as follows:

1. What is the preference of method and procedures for tracheostomy decannulation in adults?
2. What is the acceptability of TD methods and procedures?

### Eligibility of research question

The study has used the Population Intervention Comparator Outcomes (PICO) framework to determine the eligibility of research question as illustrated in Table 1 below.

**Table 1:** PICO Framework

Criteria	Determinants
Population	Adults with tracheostomies
Intervention	Tracheostomy decannulation
Comparison	Absence of TD
Outcomes	Primary—evidence for procedures and methods Secondary—preference and acceptability: successful decannulation, reduced complications of long-term tracheostomy and increased comfort by health care workers in undertaking the procedure

### Identifying relevant studies

Primary studies with a clear empirical base utilising qualitative, quantitative and mixed methods published in peer-reviewed journals as well as in grey literature addressing the research question will be included. An electronic search of the following databases will be conducted: PubMed/MEDLINE, Google Scholar, Union Catalogue of Theses and Dissertations (UCTD) via SABINET Online and World Cat Dissertations and Theses via OCLC. Websites such as the World Health Organisation (WHO) and governmental websites will be searched for policies and guidelines for TD. Studies will be identified by searching literature published in any language from January 1985 to date. A hand search through the main published texts used in otorhinolaryngology teaching and practise will also be conducted.

Articles will also be searched through the ‘Cited by’ search as well as citations included in the reference lists of included articles. The search terms will include tracheostomy, decannulation, weaning, procedures, methods, complications and adults. After searching, duplicates will be removed and the studies will be screened against the inclusion and exclusion criteria.

### Study selection

The eligibility criteria were developed to ensure specific information relating to the research question is included in the studies.

### Inclusion criteria

For studies to be included they should meet the following criteria:

- ➢ Be in all languages
- ➢ Be available in full text
- ➢ Must focus or include adult TD methods and procedures regardless of patient groups
- ➢ Must show evidence of preference and acceptability of TD methods and procedures
- ➢ Must have been published between 1985 to present

### Exclusion criteria

Studies will be excluded if they met the following characteristics

- ➢ Studies including or focusing on paediatric TD methods and procedures
- ➢ Studies published before 1985
- ➢ Studies with no evidence of preference and acceptability of TD methods and procedures
- ➢ Studies not focusing or including TD methods and procedures regardless of patient groups
- ➢ Studies not available in full text

Search strategy was piloted to check the appropriateness of selected electronic databases and key words (Table 2). An Endnote library will be created for this review. The primary investigator will conduct a comprehensive search and screening of the study titles from the above-mentioned databases. All studies with eligible titles will be exported to the endnote library, and all duplicates will be removed before abstract screening. Two reviewers will conduct abstract followed by full article screening of selected studies independently with guidance from the eligibility criteria.

**Table 2:** Pilot database search results

Keyword search	Date of search	Search engine used	Number of publications retrieved
((((((((((((((((((((((((((((((((((((((((tracheostomy[MeSH Terms]) OR tracheostomy) AND decannulation) OR tracheostomy) AND percutaneous) OR tracheostomy decannulation) OR tracheostomy care) OR tracheostomy) AND care) OR tracheostomy decannulation protocol) OR tracheostomy) AND protocol) OR tracheostomy decannulation methods) OR tracheostomy) AND decannulation methods) OR tracheostomy complications) OR tracheostomy) AND complications) OR tracheostomy weaning) OR tracheostomy) AND weaning) OR tracheostomy long stay) OR tracheostomy) AND long stay) OR tracheostomy rehabilitation) OR tracheostomy) AND rehabilitation)) OR percutaneous tracheostomy) OR percutaneous) AND tracheostomy)) OR removal tracheostomy) OR removal) AND tracheostomy)) OR decannulation method) OR decannulation) AND method)) AND ("1985/01/01"[Date - Publication] : "3000"[Date - Publication])	18 June 2017	Medline via Pubmed	2550

To optimise the article search procedure, we will utilise our local library services, the UKZN library services to help with retrieving and finding articles to be included in the full-article screening. Authors will also be contacted for electronically unavailable papers.

**Fig. 1:**
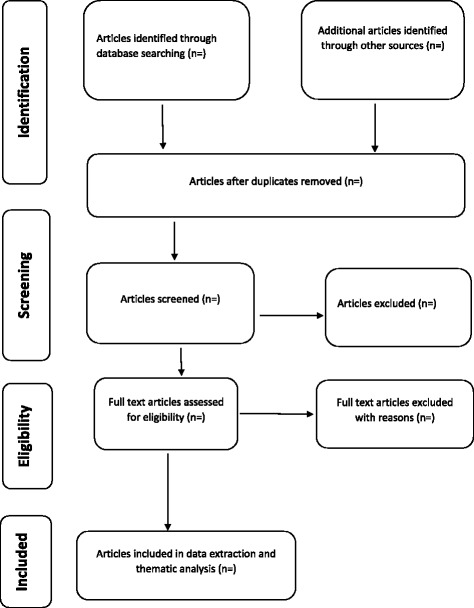
PRISMA flow chart demonstrating literature search and selection of studies

The screening results will be reported by use of the adapted PRISMA chart as in Fig. 1.

### Charting of data

Data charting table (Table 3 below) will be used to extract background information and process the information from each utilised study. A data charting form highlighting the important aspects for the study will be developed and piloted. The variables and themes included will answer the research question. The data charting form will be continually updated.

**Table 3:** Data charting table form

Author and date
Journal full reference
Aims or research questions
Recruitment context/study population
Sampling method
Study design
Theoretical background
Data collection methods used
Data analysis employed
Intervention (TD)
Intervention outcomes (methods and procedures: evaluation, removal and monitoring; preference, acceptability)
Most relevant finding
Level of evidence
Conclusions
Comments

### Collating, summarising and reporting the results

A narrative account of the data extracted from the included studies will be analysed using the thematic content analysis. Data will be extracted around the following themes: TD procedures and methods, utility and acceptability, reduced complications of long-term tracheostomy, complexity of intervention and comfort by health care workers in undertaking the procedure. Emerging themes will also be extracted. The NVIVO software version 10 will be used to code and analyse data from included studies.

### Synthesis

The resulting themes will be analysed and their relationship to the research question critically examined. Reviewers will also analyse the implication of the findings in relation to the aim of the study as well as to future research and evidential framework for policy and practise in low- and medium-income settings. An attempt will be made to draw from evidence safe and effective guidelines that are practical in low- to medium-income countries for specific patient groups.

### Quality appraisal

The Mixed Method Appraisal Tool (MMAT)-version 2011 [20] will be used to determine quality of the studies. For appraising a qualitative study, we will use section 1 of the MMAT, for a quantitative study, we will use section 2 for randomised controlled, section 3 for non-randomised, and section 4 for descriptive studies. For a mixed methods study, we will use section 1 for appraising the qualitative component, the appropriate section for the quantitative component (2 or 3 or 4) and section 5 for the mixed methods component. The tool will be used to examine the appropriateness of aim of study, adequacy and methodology, study design, data collection, study selection, data analysis, presentation of findings, author’s discussions and conclusions. The results from scrutiny of above mentioned aspects will determine quality of resultant article.

## Discussion

Tracheostomy decannulation marks a significant point in-patient rehabilitation post a frequently severe illness. It marks the return to normal or near normal phonation with improved communication, improved physical appearance and elimination of potential health complication of having a tracheostomy. A recent systematic review by Santus et al. [21] focussed on assessing predictor factors of successful decannulation and to propose a predictive score to help clinicians in choosing decannulation timing. Another more recent systematic review by Singh et al. [10] focussed on objective criteria for decannulation. Both studies concluded there is need for higher evidence research around the subject; we however aim to map literature around the whole process, contextualise it according to its themes to allow a better understanding thereby exposing knowledge deficits from which the higher evidence research can be built on.

Tracheostomy in children is different from tracheostomy in adults in terms of indications and decannulation time although the complications are similar [22]. This systematic scoping review focuses on decannulation in adults regardless of the indication for tracheostomy. It includes all studies from January 1985 to date because studies published prior are unlikely to reflect or include aspects pertaining to percutaneous dilatational tracheostomy which was first published in that year. It is anticipated that the results of this systematic scoping review will contribute to safe and effective rehabilitation of patients with tracheostomies.

## Abbreviations

ICU: Intensive care unit
MMAT: Mixed Method Appraisal Tool
PICO: Population Intervention Comparator Outcomes
PRISMA: Preferred Reporting Items for Systematic Reviews and Meta-Analyses
TD: Tracheostomy decannulation
